# Metal Body Burden as Cardiovascular Risk Factor in Adults with Metabolic Syndrome and Overweight–Obesity Analysed with an Artificial Neural Network: The Role of Hair Mineralograms

**DOI:** 10.3390/metabo13060679

**Published:** 2023-05-23

**Authors:** Luisella Vigna, Amedea Silvia Tirelli, Enzo Grossi, Stefano Turolo, Laura Tomaino

**Affiliations:** 1 Occupational Health Unit, Fondazione IRCCS Ca’ Granda, Ospedale Maggiore Policlinico, 20122 Milan, Italy; 2 Villa Santa Maria Foundation, 22038 Tavernerio, Italy; 3 Pediatric Nephrology and Dialysis, Fondazione IRCCS Ca’ Granda Ospedale Maggiore Policlinico, 20122 Milan, Italy; 4 Emergency Medicine Residency Program, Università Politecnica delle Marche, 60126 Ancona, Italy; 5 Department of Clinical and Molecular Sciences, Università Politecnica delle Marche, 60020 Ancona, Italy

**Keywords:** cardiovascular disease, obesity, metabolic syndrome, hair mineralogram, artificial neural networks

## Abstract

In determining the so-called “body burden”, hair has been widely accepted for assessing toxic element exposure. However, its role in assessing essential elements is controversial. This study investigates the possible relationship between hair minerals, metabolic syndrome (MetS) and cardiovascular (CV) risk in non-occupationally exposed subjects with overweight–obesity. Ninety-five voluntary participants (aged 51 ± 12) were recruited in Northern Italy. Hair samples were collected and analysed via inductively coupled plasma mass spectrometry; the total toxicity index (TI) was calculated as well. To evaluate cardiovascular risk factors in the presence or absence of MetS, the following factors were considered via the innovative artificial neural network (ANN) method Auto-CM: hair mineralograms (31 elements) and 25 variables including blood pressure, anthropometric parameters, insulin resistance and biochemical serum markers assessing inflammation. The Framingham risk score, fatty liver index (FLI), visceral adiposity index and CV risk scores were also taken into consideration. As shown by the semantic map, which was subsequently confirmed by an activation and competition system (ACS), obesity parameters are strictly associated with CV risk factors, TI and inflammation; meanwhile, the single mineral elements seem to be unimportant. Data obtained via ANN demonstrate that MetS may be at least partly mediated by altered mineral levels also in the presence of obesity and that waist circumference is a crucial point to be monitored rather than BMI alone. Furthermore, the mineral body burden is one of the important factors for CV risk.

## 1. Introduction

Over the last few decades, there has been an increasing interest in studying the consequences of exposure to exogenous elements because it has several health effects, such as developmental disorders, endocrine disruption, immunological syndromes, different types of cancers and even death [1]. Although human exposure to exogenous elements is often occupational due to the high level of industrial use of these elements, attention is moving towards non-occupational environments because potentially harmful metals such as Lead (Pb), Cadmium (Cd), Nickel (Ni), Arsenic (As) and Uranium (U) are contained in particulate matter and in soil [2].

The contamination of fresh water by As is an increasingly important problem since it has a well-known carcinogenic effect. Similarly, Pb has detrimental effects on the development of the nervous system, and the findings of recent studies regarding its impact on humans show that it is impossible to indicate a safe level of exposure. Cd contamination in farming soil leads to exposure through the consumption of polluted vegetables. The consequences of cumulative and long-term exposure to mixtures of metals of different origins, called the “cocktail effect”, are somehow underestimated [3]. Moreover, acute and chronic exposure to air pollution and particulate matter can be associated with premature deaths, which are mainly caused by cardiovascular (CV) diseases [4].

Obesity represents a factor of susceptibility to the adverse CV effects of pollution through different possible mechanisms. An increase in the body mass index (BMI) correlates with an increase in particulate absorption [5], and obesity itself modifies the cardiac autonomic response (in terms of heart rate variability) to particulate matter (PM) [6]. Moreover, the vascular inflammation response to PM_2.5_, which occurs through C-reactive protein mediation, is greater in subjects with obesity [7]. Some evidence shows that an increase in BMI correlates with higher deposits of fine particles in the lungs [8]. A positive correlation between exhaled nitric oxide, a marker of lung inflammation, and BMI was observed in healthy adult subjects [9].

The existing data are still contradictory, despite the presence of multiple studies indicating altered trace element status in obesity. Evidence is lacking as to the direction of causality between these factors. The gap could be addressed by means of an artificial neural network (ANN) analysis and an innovative data mining analysis known as an auto-contractive map (Auto-CM), which is based on an ANN architecture. Auto-CM allows for the discovery of hidden trends and associations among variables via a fuzzy clustering approach [10]. The added value of this approach is represented by its ability to evidence the organizing principles of a network of variables, which allows for the mapping of biological processes and the use of automatic and analytical models to reconstruct the imprecise, non-linear and simultaneous pathways underlying a complex set of data. In the last decade, Auto-CM has been successfully tested in the medical field as well [11].

The aim of the present study is to find possible hidden trends and associations between hair minerals, metabolic syndrome (MetS) and CV risk in non-occupational exposed subjects with overweight–obesity via ANN methods.

## 2. Materials and Methods

### 2.1. Participants

During a workers’ health promotion campaign for reducing cardiometabolic risks and a nutritional educational program, 95 non-occupationally exposed participants with overweight–obesity were recruited at the Centro Obesità e Lavoro, Fondazione IRCCS Ca’ Granda, Ospedale Maggiore Policlinico, Milan, Italy. Upon entering the study, each participant signed an informed consent form and underwent a medical examination. The exclusion criteria was a history of current chronic or neoplastic disease. The study was approved by the Ethics Committee of Milan Policlinico Hospital (study registration number: 1370). We used people-first language (according to the recommendation of the European Association for the Study of Obesity, EASO) to reduce bias associated with the term “obesity” and to stop stigma that labels patients by their condition [12].

### 2.2. Anthropometrics and Lab Tests

Weight, height, waist circumference (WC) and body mass index (BMI) were obtained via anthropometric evaluations. The BMI was calculated as the ratio between the weight (kg) and height (m^2^), and obesity was defined for a BMI > 30 Kg/m^2^. Systolic and diastolic blood pressure (SBP and DBP, respectively) were measured, and the mean was obtained from three measurements taken at 3-minute intervals with a conventional sphygmomanometer and the patient in a supine position. 

Blood fasting tests were performed to measure glucose, insulin, glycated haemoglobin (HbA1c), creatinine, total cholesterol (T-chol), LDL cholesterol (c-LDL), triglycerides (TG), uric acid (UA), fibrinogen, homocysteine (Hcy), highly sensitive C-reactive protein (CRP) and total blood count. Biochemical parameters were assessed using routine laboratory methods on a Modular P automated analyser (Hitachi-Roche, Basel, Switzerland) with the relevant reference intervals or cut-offs currently used in our routine laboratory. The quantitative determination of fibrinogen in citrate plasma samples was achieved using an automated I.L. Coagulation System (Instrumentation Laboratory), and HbA1c was measured via high-pressure liquid chromatography (HPLC) on a VARIANT II Turbo Instrument (BIORAD Italia, Segrate, Italy). A total blood count was performed on a Sysmex XE-2100 haematology analyser (Dasit Italia, Cornaredo, Italy).

### 2.3. Indexes

For the present study, the following indexes were calculated. Metabolic syndrome was diagnosed according to the 2005 US National Cholesterol Education Program—Adult Treatment Panel III criteria (NCEP ATP III) [13]. The Framingham risk score [14], CV risk, as described in “Progetto Cuore” [15], the visceral adiposity index (VAI) [16], and the fatty liver index (FLI), described elsewhere [17], were calculated for each participant as well. In particular, the VAI was determined on the basis of BMI, WC, TG and HDL cholesterol levels. It is indicative of adipose distribution and thus of cardiometabolic risk [18]. Reference values were defined according to age and had a range of <2.23 for adults aged 30 to 41 years, <1.92 for those between 42 and 51 years old, <1.93 for those aged 52 to 65 years, and <2 for adults aged ≥66 years [19]. On the other hand, the FLI is an algorithm based on the BMI, WC, TG and gamma-glutamyl-transferase that was developed almost a decade ago to predict fatty liver in the general population. The index ranges from 0 to 100 in which an FLI < 30 excludes fatty liver and an FLI ≥ 60 points to it [20]. The above cut-off indexes were used by clinicians but were not in the present ANN analysis.

### 2.4. Hair Analisys

All hair specimens were cut within 3 cm from the scalp and stored in labelled Ziploc bags at room temperature. The samples were mailed to Doctor’s Data in the individual kits provided and were treated using their laboratory analysis protocols [21]. They were analysed for metal content using inductively coupled plasma mass spectrometry (ICP-MS) as described elsewhere [22]. The metals assessed with this technique were: Aluminium (Al), Antimony (Sb), Arsenic (As), Barium (Ba), Bismuth (Bi), Cadmium (Cd), Lead (Pb), Mercury (Hg), Uranium (U), Nickel (Ni), Silver (Ag), Tin (Sn), Titanium (Ti), Copper (Cu), Zinc (Zn), Manganese (Mn), Chrome (Cr), Vanadium (V), Molybdenum (Mo), Boron (B), Iodine (I), Lithium (Li), Selenium (Se), Strontium (Sr), Sulphur (S), Cobalt (Co), Iron (Fe), Germanium (Ge), Rubidium (Rb), Zirconium (Zr) and Gold (Au). A total toxic element score, or toxicity index (TI), was calculated by the Doctor’s Data laboratory using a weight average based upon the relative toxicity (per gram) of individual elements. The higher the TI score, the more toxic the combination of hair metals.

### 2.5. ANN Analysis

The use of standard statistical models is difficult because many relationships in the system are unknown. The use of an artificial neural network is suitable for constructing a good model as it can implicitly take into account all dependencies in the system and process inaccurate and incomplete data. Therefore, in this study, data were analysed using an ANN with the Auto-Contractive Map (Auto-CM) software, developed by Semeion Research Center Science Communication (Rome, Italy) and described elsewhere [11]. All the parameters considered were processed simultaneously. Through this method, correlations were made, non-linear associations between variables were maintained and connection patterns between groups of variables were captured at the same time. The result is a map with nodes (important points) and arcs (connections) which must be interpreted according to the available evidence. The system also provides a quantification of the “strength” of the links among variables (nodes of the graph) via a numerical coefficient, called the link strength, which ranges from 0 (minimum strength) to 1 (maximum strength). The superimposed value is proportional to the strength of the link and can be read as the probability of a transition from any state variable to any other state variable [23]. To run this model, dichotomic values were obtained for each of the variables considered in the present study. This pre-processing scaling allowed for a proportional comparison among all the variables and implied the existence of links of each variable when the values tended to be high or low. Finally, the data were processed using an auto-associative neural network developed by Buscema et al. [24] known as the activation and competition system (ACS), (application described elsewhere [25] and in Appendix A) in order to emphasize the causal relationship between TI and CV risk, as expressed by the Framingham risk score. We have deliberately avoided pruning since the approach with this unusual data set was free of a hypothesis. The ultimate goal of this data mining model is to discover hidden trends and associations among variables since this algorithm is able to create a semantic connectivity map in which non-linear associations are preserved, and explicit connection schemes are described. In Appendix A, the detailed methods of Auto-CM and ACS are reported.

## 3. Results

The present study used the hair mineralogram as an indicator of exposure and the internal dose of the different toxic metals in order to assess the possible relation to MetS and CV risk in our group of subjects with overweight–obesity. Table 1 is a description of the study sample, which consisted of 95 participants (aged 51 ± 12 years).

Table 2 provides the mean contents in terms of the minerals and toxic elements from the hair samples of the 95 participants. Most of the toxic elements were above the reference values. Of note: the Barium (Ba) level was (mean ± SD) 1.29 ± 1.63 μg/g (versus a reference value of <0.75), Cadmium (Cd) was 0.74 ± 6.66 μg/g (reference value < 0.070), the Lead (Pb) level was 1.59 ± 3.49 μg/g (versus a reference value < 1.0), Mercury (Hg) was 1.64 ± 1.49 μg/g (reference value < 0.40), Uranium (U) was 0.11 ± 0.12 μg/g (versus < 0.060) and Nickel (Ni) was 0.52 ± 0.82 μg/g (normal range < 0.20). Finally, Silver (Ag) was 1.68 ± 5.13 μg/g (normal value < 0.14) and Tin (Sn) was 0.86 ± 1.48 μg/g (normal value < 0.30). Regarding the hair minerals, most of the elements fell within the range of normality with the exception of Strontium (Sr), which had a mean value of 9.70 ± 8.16 μg/g (versus a reference value of 0.21–2.1), and Cobalt (Co) at 0.05 ± 0.09 μg/g (versus a reference value of 0.004–0.020).

**Figure 1:**
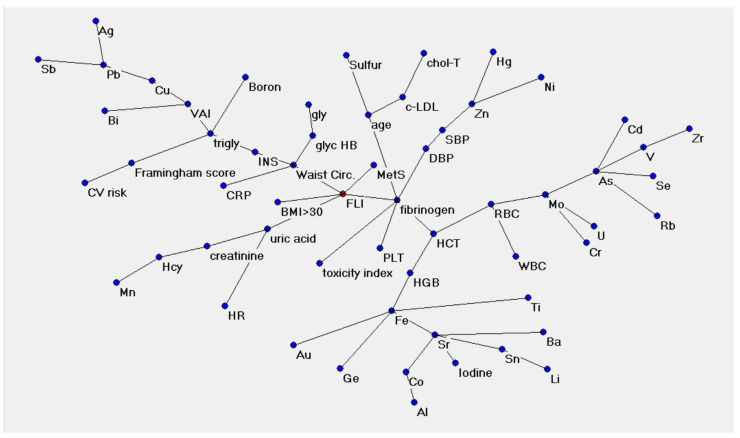
Semantic connectivity map of variables created by the Auto-CM algorithm. Abbreviations (in alphabetical order): Ag: Silver; Al: Aluminium; As: Arsenic; Au: Gold; Ba: Barium; Bi: Bismuth; Cd: Cadmium; chol-T: total cholesterol; Co: Cobalt; Cr: Chromium; CRP: C-reactive protein; Cu: Copper; CV: cardiovascular; DBP: diastolic blood pressure; Fe: Iron; FLI: fatty liver index; Ge: Germanium; gly: glucose; glycHB: glycated haemoglobin; Hcy: haematocrit; HCT: haematocrit; Hg: Mercury; HGB: haemoglobin; HR: heart rate; INS: insulin; LDL-c: LDL cholesterol; Li: Lithium; MetS: metabolic syndrome; Mn: Manganese; Mo: Molybdenum; Ni: Nickel; Pb: Lead; PLT: platelets; RBCs: red blood cells; Rb: Rubidium; Sb: Antimony; SBP: systolic blood pressure; Se: Selenium; Sn: Tin; Sr: Strontium; Ti: Titanium; Trigly: triglycerides; U: Uranium; V: Vanadium; VAI: visceral adiposity index; WBC: white blood cells; Zn: Zinc; Zr: Zirconium.

**Figure 2:**
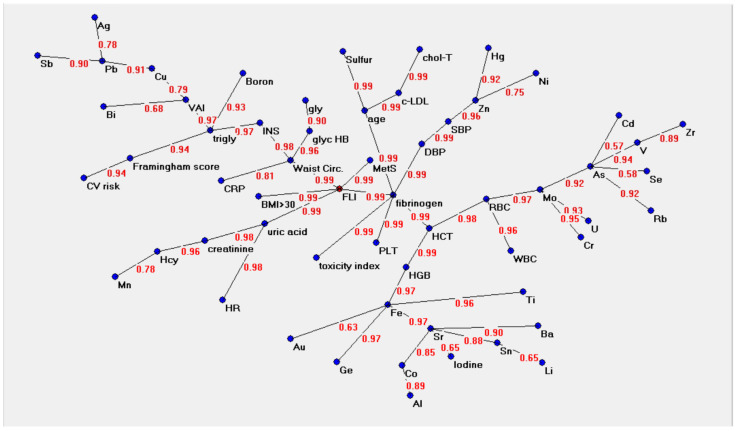
Semantic connectivity map and strength of the associations between variables created by the Auto CM algorithm. Abbreviations (in alphabetical order): Ag: Silver; Al: Aluminium; As: Arsenic; Au: Gold; Ba: Barium; Bi: Bismuth; Cd: Cadmium; chol-T: total cholesterol; Co: Cobalt; Cr: Chromium; CRP: C-reactive protein; Cu: Copper; CV: cardiovascular; DBP: diastolic blood pressure; Fe: Iron; FLI: fatty liver index; Ge: Germanium; gly: glucose; glycHB: glycated haemoglobin; Hcy: homocysteine; HCT: haematocrit; Hg: Mercury; HGB: haemoglobin; HR: heart rate; INS: insulin; LDL-c: LDL cholesterol; Li: Lithium; MetS: metabolic syndrome; Mn: Manganese; Mo: Molybdenum; Ni: Nickel; Pb: Lead; PLT: platelets; RBC: red blood cells; Rb: Rubidium; Sb: Antimony; SBP: systolic blood pressure; Se: Selenium; Sn: Tin; Sr: Strontium; Ti: Titanium; Trigly: triglycerides; U: Uranium; V: Vanadium; VAI: Visceral Adiposity Index; WBC: white blood cells; Zn: Zinc; Zr: Zirconium.

**Figure 3:**
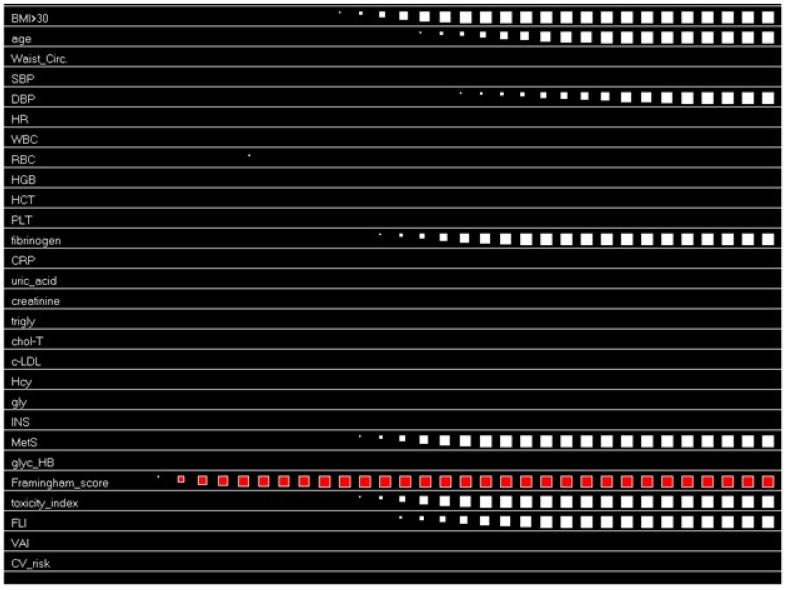
The activation and competition system (ACS) and the steady state reached after the activation of Framingham risk score. The analysis via the ACS system was performed after pruning the mineral values. Framingham risk score is hierarchically connected with a BMI > 30, TI, MetS, fibrinogen, FLI, age and diastolic blood pressure.

Figure 1 shows the semantic connectivity map of the variables which was created by the Auto-CM algorithm. The representations indicate nodes and arc connections, highlighting the links between different variables. There is a value hierarchy: nodes that have more connections are more important. The so called “minimum spanning tree” was obtained from a matrix of distances in which each node is related to the others; the smaller the distances between nodes, the higher the affinity between them. Connected variables are called a “hub”, and the variable in red (FLI in Figure 1, Figure 2 and Figure 3) is the central point of the graph.

As shown in Figure 2, the numbers along the lines (arches) refer to the strength of the association between two adjacent nodes. The range of this value is from 0 to 1. This value derives from the original weight developed by Auto-CM during the training phase; it ranges from 0 to 1 and is proportional to the strength of the connections between two variables. In the semantic map, the central hub is represented by the FLI, linked with a strength of 0.99 to the WC, MetS, BMI > 30 Kg/m^2^ (unsurprisingly, as the index is based on waist circumference, BMI, triglycerides and gamma-glutamyl-transferase) but also with fibrinogen and uric acid, as shown in Figure 2. In turn, fibrinogen is strictly connected (0.99) with platelets, age, diastolic blood pressure, haematocrit and the hair toxicity index.

WC is a hub, triply connected with glycated haemoglobin (with a strength of 0.96), which is in turn connected to blood glucose, with CRP (0.81), and with insulin, which is connected to triglycerides.

Triglycerides, then, are a hub between CV risk and the VAI. Peripherally, we can observe the branch of other CV risk factors and the Framingham risk score, which is connected to triglycerides with a strength of 0.94.

Hair minerals appear on the outskirts of the semantic map, connected in clusters to metabolic factors. VAI is the connection node for Bismuth (Bi), Copper (Cu), Lead (Pb), Silver (Ag) and Antimony (Sb). Triglycerides are linked to Boron (B) with a strength of 0.93. Age represents a hub strongly bonded (0.99) with LDL cholesterol and total cholesterol on one side and to Sulphur (S) on the other. Blood pressure, both systolic and diastolic, is connected to Zinc (Zn), Mercury (Hg) and Nickel (Ni).

Finally, all the components of the blood count bind in various ways to many hair metals and toxic elements with strengths ranging from 0.97 to 0.57, depending on the element considered.

Figure 3 graphically shows the results of the application of the ACS in evaluating the causation links between cardiovascular risk (expressed by the Framingham risk score, bwith CV risk similarly calculated by “Progetto Cuore”) and the selected variables. The activation variable is represented in red, while the response variables are in white in terms of timing and intensity. Of note, a high Framingham risk score is causally connected with a BMI > 30, age, diastolic blood pressure, fibrinogen, MetS, FLI and TI. The hierarchy in which these variables influence the activation variables is shown in the following image.

**Figure 4:**
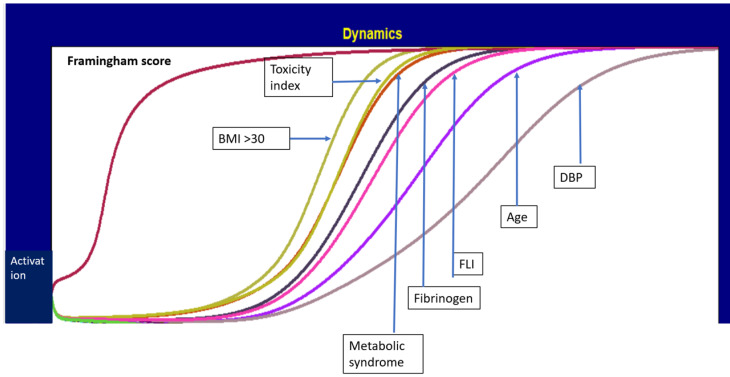
Dynamics of the variables after the activation of the Framingham risk score, leading to the steady state.

Figure 4 shows the dynamics of the variables after the activation of the Framingham index, which leads to the steady state of the ACS. This allows for a better appreciation of the hierarchy of the variables involved. In particular, the increase in the toxicity index immediately occurs after that of obesity and is practically synchronous with MetS, which is very interesting. Then, in the activation dynamics, other variables take place in the following order: fibrinogen, FLI, age and diastolic blood pressure.

## 4. Discussion

The findings of the present study show that CV risk, as expressed by the Framingham score, is strongly and unsurprisingly related to obesity (BMI > 30 Kg/m^2^) but also to the overall effect of the toxic minerals found in the hair, known as the toxicity index. 

The epidemic of CV risks and immunological and neurological diseases are likely associated with environmental toxins [26,27], which can elicit independent, additive or synergistic toxic effects. Minimal risk levels (MRLs) for exposures, i.e., the amount of a substance a person can be exposed to without a detectable health risk, have not yet been considered; humans bioaccumulate metals [28], but our knowledge of adverse effects is primarily based on studies of single toxicants. Moreover, individuals differ considerably in their sensitivities to metals, and susceptibility to toxicity varies with age, gender, pregnancy status, nutritional status, total toxic load and genetics (e.g., methylation). The potentially toxic elements differ with respect to their relative toxicities, and low-level exposures are associated with long-term effects that were previously unrecognized [29]. The accumulation of more than one toxic element may have synergistic adverse effects, even if the level of each individual element is not very high. Therefore, a total toxic element “score” was used in this study (here called toxicity index) and was estimated using a weighted average based upon the relative toxicity.

Regarding its contained elements, hair is essentially an excretory tissue rather than a functional one. It is a protein (keratine) synthesized in the hair follicle whose secreted elements are permanently incorporated into its structure. Therefore, hair element analysis provides important information owing the characteristic of a “temporal record” of element metabolism, such as Magnesium (Mg), Chromium (Cr), Zinc (Zn), Copper (Cu) and Selenium (Se), all co-factors for several enzymes and biochemical reactions and exposure to toxic substances. Hence, hair can be considered a useful, cheap and non-invasive tool for detecting recent exposure to toxic elements such as Arsenic (As), Aluminium (Al), Cadmium (Cd), Lead (Pb), Antimony (Sb) and Mercury (Hg). Nevertheless, the correlation between levels of toxic and trace elements in hair, blood or plasma is still under debate, as is the accountability of these tests [30]. For the same reason, the association between the hair concentration of selected elements and diseases, especially CV ones, remains controversial [31,32]. In spite of this, thanks to technological improvements, instrumentation and the application of scientific protocols, hair element analysis has become a valuable tool for providing reliable and useful data and may be considered an important material for biological monitoring [33]. In this scope, a toxicity index of overall exposure to metals and toxic elements may offer important information regarding biological hazards and health risks.

Metabolic syndrome (MetS) is a complex disorder defined by a cluster of interconnected factors increasing the risk of cardiovascular atherosclerotic diseases. It has been already pointed out that a high mercury concentration in hair tissue may increase the risk of metabolic syndrome [34]. A recent work concluded that trace element and mineral status may partially contribute to metabolic risk in subjects with obesity [35]. Fibrinogen, as a factor of tissue injury and inflammation [36,37], has already been described and is in line with the findings of the present study, which demonstrates fibrinogen as a hub linked to the FLI and the toxicity index. Similarly, and widely recognized among CV risk factors [38,39,40] in the ANN of the present study, waist circumference is linked with the FLI on one side and is connected on the other with the branch of the Framingham risk score and glucose metabolism. Some evidence suggests that metabolic disorders, such as obesity and type 2 diabetes, are associated with mineral disbalances, as assessed through hair mineralograms. In particular, a study analysing the hair contents of Chromium (Cr), Iron (Fe) and Zinc (Zn) in men with obesity (BMI > 30 kg/m^2^), as well as overweight and normal-weight men, observed that those with a BMI > 30 kg/m^2^ presented a mean Chromium (Cr) concentration of 0.096 μg/g and a mean Iron (Fe) concentration of 9.42 μg/g, values that were significantly higher than those of the overweight subjects, and Zinc (Zn) mean values of 183 μg/g, which were significantly lower than those of the overweight participants [41]. The findings of a similar case–control study showed reduced hair levels of Copper (Cu) and Zinc (Zn) in subjects with obesity compared to normal-weight subjects [42]. The findings of our study are in line with these results: in our sample, the values of Chromium (Cr) and Iron (Fe) were higher (0.47 and 15.7 μg/g, respectively), and Zinc (Zn) was 176.6 μg/g, while falling within the reference intervals. Another work analysed the levels of selected elements in hair samples of subjects with type 2 diabetes compared to healthy controls [43]. The findings showed that levels of Zinc (Zn), Copper (Cu) and Chromium (Cr) in the diabetic participants (with HbA1c >7) were markedly lower than in controls, and that the concentrations decreased considerably with an increase in glycated haemoglobin. The levels of Iron (Fe) and Magnesium (Mg) in the diabetic subjects were lower (though not significantly), and the concentration of Iron (Fe) decreased significantly with increases in HbA1c (*p* < 0.05). In contrast, the concentration of Arsenic (As) tended to increase with increases in HbA1c (*p* < 0.10) [43].

Deficiencies in trace elements such as Cr, Zn, Cu and Mg have been associated with glucose tolerance and insulin resistance [44,45]. For instance, the glucose tolerance factor (GTF) is a Chromium (Cr)-containing compound and is related to glucose homeostasis. The precise biochemical basis for the effect of Cr on glucose homeostasis is unknown, although some evidence suggests that GTF enhances the binding of insulin to its receptors [44]. Zinc (Zn) plays a role in insulin synthesis, stabilizes the insulin stored in pancreatic beta cells and has an important role in secreting it from the same cells [43,44,45,46]. Finally, Magnesium (Mg) is responsible for the uptake of glucose in insulin-dependent tissues.

In this study, trace elements remained at the periphery of the semantic map, suggesting a possible synergistic effect provided by a non-algebraic relationship between the single elements. For example, Copper (Cu) lies far from the centre of the map, weakly linked to the VAI, which is a useful tool in daily clinical practice and in population studies for the assessment of cardiometabolic risk associated with visceral obesity.

Lead (Pb), a toxic metal associated with adverse cardiovascular outcomes [47], is far from the hub. The same map indicates known links between minerals and biochemical parameters. It also shows that Chromium (Cr) and other toxic minerals are linked to Molybdenum (Mo), which, in turn, is linked to red blood cells and haematocrit, as it is known that Molybdenum enhances the osmotic resistance of red blood cells [48]. Iron, which binds to various other metals, is known to correlate with haemoglobin and haematocrit. Boron, linked with triglycerides, may act as a metabolic regulator in several enzymatic systems, as has been proven by research on animal models.

Zinc (Zn) is one of the essential trace elements whose impact on hypertension is documented. As a micronutrient, it functions as a co-factor for up to 10% of the proteins in living organisms, playing a vital role in a range of biological processes in the human body [49]. In the semantic map of this study, Zn is related to blood pressure and, in turn, to fibrinogen. Additionally, toxic elements lie on the outskirts of the semantic map, while surprisingly, the toxicity index is strictly linked to the fibrinogen hub. To explain the increase in the amount of reactive oxygen species (ROS) due to hyperglycaemia, we should take into account a possible link between complications of MetS and alterations in the hair mineral content [50]. Superoxide dismutase (SOD) nullifies the effects of superoxide by converting it into hydrogen peroxide, Zn and Cu act as cofactors for the isomers of the SOD enzyme [51]. Fibrinogen has already been indicated as an altered inflammatory index parameter in subjects with metabolic syndrome and obesity [52].

The findings of this study and especially of the ACS show that CV risk, expressed through the Framingham score, is strictly related to obesity and the toxicity index. It is also practically synchronic to MetS in already known parameters such as age and diastolic blood pressure and in less-known parameters such as fibrinogen and FLI. In a recent retrospective study, FLI was indicated not only as a predictor of a non-alcoholic fatty liver disease diagnosis but also as designating baseline for the future development of cardiovascular disease on long-term follow-ups across weight categories and the fatty liver index spectrum [53]. Lastly, in calculating the FLI, waist circumference is taken into consideration and BMI is not. This underlines the importance of adding it to simple parameters, clinically determining the risk of CVD in subjects with obesity and with Mets. The innovative data analysis with the ANN is one of the strengths of the present study because the ANN can simultaneously process all the parameters considered and investigate all linear and non-linear relationships within them.

The limitation of the study design is the absence of a normal-weight control group; therefore, we chose neural networks to analyse the database, as they could possibly highlight the hidden link between the considered variables. As already stated in the introduction [1,2,3,4] and recently reported by the available literature [47,54], toxic metals (i.e., Lead, Arsenic and Cadmium, among others) contribute to an increased CVD risk, with environmental contamination a major concern in need of further investigation and monitoring. Hair is potentially an excellent marker of exposure to metals [55]; unfortunately, it has a set of limitations that mean it is not always reliable, as it is affected by different factors that are also specific to certain regions and subjects.

We should also bear in mind the wide dissemination of metals in the past century which affect millions of people. We feel sure that in the future, new factors will likely be reported for the effective interpretation, validity and application of results of hair analyses.

In conclusion, this study suggests the potential role of the mineral body burden on the increase in metabolic syndrome, although further studies with more subjects divided by age, gender, normal weight and pathology are needed rather than taking only subjects with overweight/obesity reacting to a single toxic mineral as a CV risk factor.

## Figures and Tables

**Table 1:** Biometrics and biochemical data of study participants.

	Unit of Measure	Mean	Standard Deviation	Minimum	Maximum	Median
BMI	-	32.7	5.7	27.5	54.7	32.1
Waist Circumference	-	99	13	75	137	97
Systolic Blood Pressure	mmHg	124	17	85	170	120
Diastolic Blood Pressure	mmHg	79	11	55	100	80
Heart rate	bpm	72	9	52	100	70
White blood cells	10 × 10^9^/L	6.90	1.79	3.69	14.39	6.67
Red blood cells	10 × 10^12^/L	4.85	0.48	3.71	6.49	4.77
Haemoglobin	g/dL	13.7	1.3	10.4	17.4	13.6
Haematocrit	%	41	3	32	50	41
Platelets	10 × 10^9^/L	248	59	72	414	247
Fibrinogen	mg/dL	332	61	70	491	328
C-reactive protein	mg/dL	0.51	0.77	0.03	4.48	0.25
Uric acid	mg/dL	4.9	1.2	1.8	8.2	4.7
Creatinine	mg/dL	0.74	0.13	0.51	1.16	0.72
Triglycerides	mg/dL	109	47	43	279	98
Total cholesterol	mg/dL	216	41	126	336	213
LDL cholesterol	mg/dL	132	36	58	226	128
Homocysteine	μmol/L	10.9	3.8	5.0	27.8	10.4
Glycaemia	mg/dL	96	27	57	297	91
Insulin	mIU/mL	12.50	6.01	0.40	32.60	11.90
Glycated haemoglobin, HbA1c	%	5.9	0.7	4.4	9.5	5.8
Fatty liver index		57.6	28.5	5.0	99.7	63.0
Visceral adiposity index		3.8	2.6	0.9	17.5	2.7
Framingham risk score		3.8	3.9	0	20	2.0
CV risk “Progetto Cuore”		4.1	5.5	0.2	29	2.1

**Table 2:** Hair mineral elements in 95 subjects.

		Reference Interval μg/g	Mean	Standard Deviation	Minimum	Maximum	Median
**Toxic metals**	Aluminium (Al)	<8.0	7.64	13.12	0.70	94.00	4.10
	Antimony (Sb)	<0.066	0.05	0.12	0.01	1.10	0.02
	Arsenic (As)	<0.080	0.04	0.04	0.01	0.27	0.03
	Barium (Ba)	<0.75	1.29	1.63	0.04	11.00	0.95
	Bismuth (Bi)	<2.0	0.06	0.18	0.00	1.60	0.02
	Cadmium (Cd)	<0.070	0.74	6.66	0.00	65.00	0.03
	Lead (Pb)	<1.0	1.59	3.49	0.04	29.00	0.59
	Mercury (Hg)	<0.40	1.64	1.49	0.09	7.80	1.20
	Uranium (U)	<0.060	0.11	0.12	0.01	0.63	0.07
	Nickel (Ni)	<0.20	0.52	0.82	0.04	6.20	0.30
	Silver (Ag)	<0.14	1.68	5.13	0.01	38.00	0.21
	Tin (Sn)	<0.30	0.86	1.48	0.02	7.80	0.28
	Titanium (Ti)	<0.70	0.74	0.44	0.24	2.40	0.59
**Essential and other elements**	Copper (Cu)	11–32	17.01	14.96	6.70	140.00	14.00
	Zinc (Zn)	110–190	176.6	63.7	61.0	440.0	170.0
	Manganese (Mn)	0.08–0.50	0.27	0.37	0.03	3.20	0.18
	Chromium (Cr)	0.40–0.70	0.47	0.10	0.33	0.94	0.44
	Vanadium (V)	0.025–0.10	0.05	0.04	0.01	0.33	0.04
	Molybdenum (Mo)	0.040–0.090	0.03	0.01	0.01	0.08	0.03
	Boron (B)	0.50–3.5	0.97	0.82	0.07	4.70	0.68
	Iodine (I)	0.25–1.3	1.14	3.06	0.07	26.00	0.51
	Lithium (Li)	0.007–0.020	0.02	0.08	0.00	0.75	0.01
	Selenium (Se)	0.70–1.1	0.94	2.54	0.21	25.00	0.62
	Strontium (Sr)	0.21–2.1	9.70	8.16	0.16	35.00	9.20
	Sulphur (S)	44,000–51,000	47,376	1955	43,400	51,400	47,400
	Cobalt (Co)	0.004–0.020	0.05	0.09	0.00	0.49	0.02
	Iron (Fe)	7.0–16	15.07	6.22	3.90	36.00	15.00
	Germanium (Ge)	0.030–0.040	0.03	0.01	0.03	0.05	0.03
	Rubidium (Rb)	0.008–0.080	0.08	0.17	0.00	1.10	0.02
	Zirconium (Zr)	0.060–0.70	0.05	0.06	0.01	0.40	0.03
	Gold (Au)	<0.50	0.14	0.34	0.00	3.00	0.05

**Figure A1:**
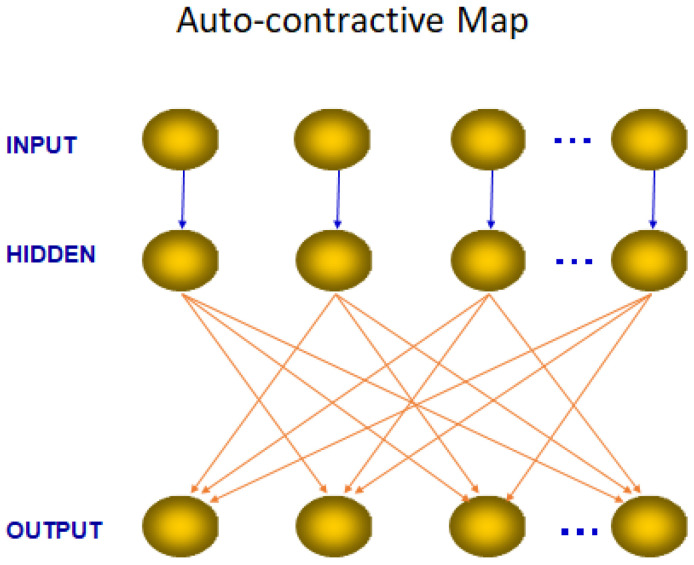
Architecture of the Auto CM neural network.

**Figure A2:**
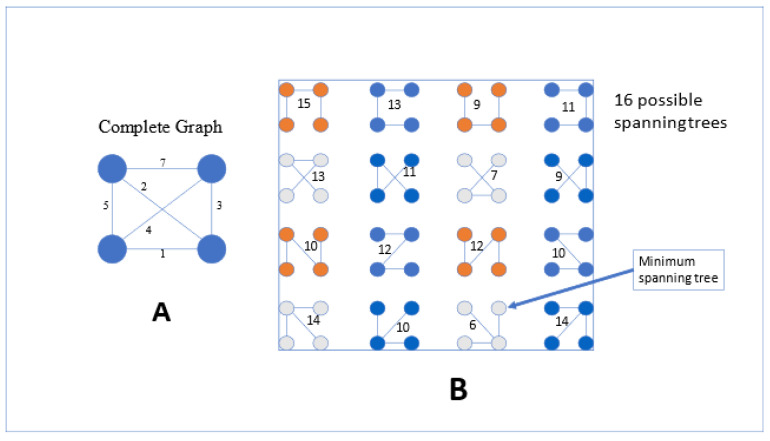
Minimum spanning tree.

## Data Availability

Data sharing not applicable. Data is not publicly available due to privacy or ethical restrictions.

## Appendix A

### Appendix A.1. Auto-CM

The auto-contractive map (Auto-CM for short) was born as a new artificial neural network. It was designed by Massimo Buscema at the Semeion Research Center. The Auto-Cm system finds, via a specific learning algorithm, a square matrix of weighted connections among the variables of any dataset. This matrix of connections presents many suitable features: (a) non-linear associations among variables are preserved; (b) connection schemes among clusters of variables are captured; and (c) complex similarities among variables become evident. The ultimate goal of this data mining model is to discover hidden trends and associations among variables since this algorithm is able to create a semantic connectivity map in which non-linear associations are preserved and explicit connection schemes are described. Briefly, this approach describes a typical context of living systems in which a continuous, time-dependent, complex change in the variable value is present. A simple filter (a minimum spanning tree by Kruskal) is then introduced into the matrix of the Auto-CM system. This approach shows the map of relevant connections between and among variables and the principal hubs of the system. Hubs can be defined as variables with the maximum number of connections in the map. The architecture of Auto-CM is shown in Figure A1.

The specificity of the Auto-CM algorithm is to minimize a complex cost function with respect to the traditional functions:

Traditional minimization cost function:

(A1) $$E=Min\left\{\overset{N}{\underset{i}{\sum }}\overset{N}{\underset{j}{\sum }}\overset{M}{\underset{q}{\sum }}u_{i}^{q}\cdot u_{j}^{q}\cdot \sigma_{i,j}\right\}$$

Auto-CM minimization cost function:

$$\begin{array}{l} E=Min\left\{\overset{N}{\underset{i}{\sum }}\overset{N}{\underset{j}{\sum }}\overset{N}{\underset{k}{\sum }}\overset{M}{\underset{q}{\sum }}u_{i}^{q}\cdot u_{j}^{q}\cdot u_{k}^{q}\cdot A_{i,j}\cdot A_{i,k}\right\};\\ A=\left(1.0-\frac{w}{C}\right);\\ N=\;\mathrm{Number}\;\mathrm{of}\;\mathrm{Variables}\;\left(\mathrm{Columns}\right);\\ M=\;\mathrm{Number}\;\mathrm{of}\;\mathrm{Patterns}\;\left(\mathrm{Rows}\right). \end{array}$$

From a mathematical point of view, it becomes evident that the traditional minimization includes only second-order effects, while Auto-CM considers also a third order. Practically, this means that the Auto-CM algorithm is able to discover variable similarities that are completely embedded into the dataset and invisible to the other classical tools. This approach describes a context which is typical of living systems in which a continuous, time-dependent, complex change in the variable value is present. After the training phase, the matrix of the Auto-CM algorithm represents the warped landscape of the dataset. The auto-contractive map is a special type of ANN which, compared with classical ANNs which have random initial weight values for the connections, begins from all connections set up with the same values. Therefore, it does not suffer the problem of the symmetric connections. During training, it develops for each connection only positive values. Therefore, Auto CM does not present inhibitory relations among nodes but only different strengths of excitatory connections. Auto CM can also learn under hard conditions, that is, when the connections of the main diagonal of the second connection matrix are removed. When the learning process is organized in this way, Auto CM seems to find a specific relationship between each variable and any other. Consequently, from an experimental point of view, it seems that the ranking of its connection matrix is equal to the ranking of the joint probability between each variable and the others.

Once an Auto-CM connection matrix is obtained, it is then filtered by a minimum spanning tree algorithm (MST), generating a graph of which biological evidence has already been tested in the medical field [10,11,56,57].

### Appendix A.2. Minimum Spanning Tree

A minimum spanning tree (MST) is a spanning tree of a connected, undirected graph. It connects all the vertices together with the minimal total weighting for its edges. The MST algorithm was originally described by the Czech scientist Otakar Boruvka in 1926, who aimed to optimize the planning of electricity connections among cities, and was later on refined by Kruskal with a specific deterministic algorithm [58].

In the bio-medical field, the MST has been used particularly in microarray clustering. Although MST-based clustering is formally equivalent to the dendrograms produced by hierarchical clustering under certain conditions, they can be extremely visually different. MST has to do with the least action principle.

In classical mechanics, Maupertuis’s principle states that the path followed by a physical system is the one of least length. It is a special case of the more generally stated principle of least action. Using the calculus of variations, it results in an integral equation formulation of the equations of motion for the system. The energy-based least action principle (LAP) has proven to be very successful for explaining natural phenomena in both classical and modern physics.

In biological systems, the kinetic paths from the least action principle quantify the transition processes among normal state and the pathological state. For this reason, our assumption is that also in case of variables describing normal and pathological states, their interconnection system must naturally tend to least length, which is described well by the graph generated by the MST. Thus, a minimum spanning tree is a spanning tree with a weight less than or equal to the weight of every other spanning tree. In practical terms, the MST shows the best way to connect the variables in a tree and the shortest possible combination, allowing to the data to be presented in a simplified graph.

The main advantage of the MST algorithm is to provide a synthetic view of the variable ensemble and to make it very easy to understand clustering through links directly connecting variables that are very close each other. The importance of the variables in the graph is related to their number of links. Hubs may be defined as the variables with the maximum number of connections in the graph. The clustering distance among two variables is related to their degrees of separation.

A single graph can have many different spanning trees. We can also assign a weight to each edge, which is a number representing how unfavourable it is, and use this to assign a weight to a spanning tree by computing the sum of the weights of the edges in that spanning tree. The minimum spanning tree (MST) displays the shortest combination out of all potential methods to connect the variables in a tree, as shown by the example in Figure A2.

Figure A2 shows the graph theory applied to four points (variables) with the distances visible on the arches in a multi-dimensional space. Part A of Figure A2 depicts a complete graph in which all points are connected. Part B of Figure A2 describes sixteen possible spanning trees, i.e., the possibilities of connecting the four points while avoiding loops. By considering the distances, there is one spanning tree in which the sum of the distances produces a shortest path (sum = 6). This is the minimum spanning tree of this set of points.
